# Potential Effects of Digoxin on Renal Functions in Patients With Congestive Heart Failure

**DOI:** 10.7759/cureus.45419

**Published:** 2023-09-17

**Authors:** Mesfer A Alqahtani, Bader A Alqahtani, Ibrahim M Dighriri

**Affiliations:** 1 Department of Medical Supply, Khamis Mushayt Maternity and Children Hospital, Khamis Mushait, SAU; 2 Department of Pharmacy, Tathleeth General Hospital, Tathleeth, SAU; 3 Department of Pharmacy, King Abdulaziz Specialist Hospital, Taif, SAU

**Keywords:** creatinine, bun, egfr, chf, renal function, digoxin

## Abstract

Introduction: Renal dysfunction is a common complication among patients with congestive heart failure (CHF) and can significantly impact their management, especially when medications like digoxin are involved. The clearance of digoxin is closely tied to the glomerular filtration rate (GFR), which suggests that the safety and efficacy of digoxin may vary with renal function. Therefore, this study aimed to assess the potential effects of digoxin on renal function in patients diagnosed with CHF at a tertiary hospital in the Asir region of Saudi Arabia.

Methods: A retrospective study examined the records of 30 CHF patients treated with digoxin. Renal function markers like estimated GFR (eGFR), creatinine, blood urea nitrogen (BUN), albumin, and urine levels were compared before and after digoxin treatment. Liver enzymes and other relevant parameters were also examined. A statistical analysis using t-tests was conducted to evaluate the changes in renal function indicators before and after digoxin treatment.

Results: The mean eGFR decreased significantly from 65.4 ± 8.9 mL/min/1.73m^2 ^before digoxin to 57.7 ± 7.8 mL/min/1.73m^2^ after (p = 0.001). Creatinine, BUN, albumin, and urine levels showed no significant changes. Digoxin significantly increased aspartate aminotransferase (AST) from 34.5 ± 11.6 U/L to 53.8 ± 14.6 U/L (p = 0.002), alanine aminotransferase (ALT) from 38.5 ± 12.6 U/L to 55.3 ± 17.6 U/L (p = 0.013), and creatine kinase from 117.7 ± 22.5 U/L to 133.9 ± 15.8 U/L (p = 0.012). Hemoglobin decreased significantly from 12.8 ± 1.4 g/dL to 12.1 ± 1.4 g/dL (p = 0.034). No significant changes occurred in myoglobin, troponin, bilirubin, platelets, potassium, calcium, or chloride levels. Effects on kidney function did not differ significantly by gender or age, except blood urea nitrogen was higher in patients over 50 years (8.3 ± 2.3 vs. 5.6 ± 2.7 mg/dL, p = 0.015).

Conclusion: This study suggests digoxin may adversely affect renal function in CHF patients, as evidenced by reduced eGFR. However, the small retrospective design limits definitive conclusions. Further prospective research with larger samples is warranted to elucidate digoxin's renal effects in CHF patients.

## Introduction

Renal dysfunction is a prevalent complication among congestive heart failure (CHF) patients [1,2]. This renal dysfunction can notably complicate heart failure (HF) therapy, especially when treatment involves digoxin, a drug primarily eliminated by the kidneys [3]. The clearance of digoxin is closely tied to the glomerular filtration rate (GFR), which suggests that the safety and efficacy of this drug may vary with renal function [3,4].

In patients with renal dysfunction, the elimination half-life of digoxin is prolonged, and systemic exposure is increased. Combined with digoxin's narrow therapeutic index, this could lead to digoxin toxicity in HF patients [4,5]. However, digoxin's inotropic effect, which increases cardiac contractility, counterbalances this potential toxicity. The enhanced perfusion of all body tissues, including the kidneys, could mitigate the kidney dysfunction associated with, or even induced by, HF [4,6].

This suggests a complex interplay between heart failure, kidney dysfunction, and digoxin [5,6]. On one hand, HF can give rise to kidney dysfunction, increasing the risk of digoxin toxicity. On the other hand, the enhanced heart contractility caused by digoxin could potentially improve kidney function [6]. Therefore, assessing this intricate relationship between digoxin and renal function is vital.

Despite the widespread use of digoxin in treating HF and atrial fibrillation, its interaction with renal function remains inadequately understood, particularly in the context of HF patients in Saudi Arabia. Renal dysfunction can elevate the risk of digoxin toxicity due to prolonged drug elimination and systemic exposure [3]; the inotropic effect of digoxin could enhance kidney perfusion and thus alleviate kidney dysfunction associated with HF [2,4]. This complex relationship between digoxin and renal function has not been sufficiently investigated in the Asir region of Saudi Arabia. This underscores the need for comprehensive studies examining this interaction among the region's CHF patients.

Therefore, this study aims to assess the potential effects of digoxin on renal function in patients diagnosed with CHF at a tertiary hospital in the Asir region, Saudi Arabia. By deepening our understanding of the interplay between digoxin and renal function, we can optimize treatment plans for HF patients with renal dysfunction. This would empower clinicians to make informed decisions, minimizing the potential risks of digoxin toxicity while maximizing its therapeutic benefits, ultimately leading to improved quality of life and clinical outcomes for patients grappling with the dual challenges of CHF and renal dysfunction.

## Materials and methods

This study adopted a retrospective clinical approach, using data from hospital records. The study was conducted in Asir Central Hospital, located in Abha, in the Asir region of Saudi Arabia. We examined the records of patients from the cardiology and nephrology departments. Patients diagnosed with CHF and treated with digoxin between July 2021 and December 2022 were included. Records of patients with prior renal diseases or those on other nephrotoxic drugs were excluded from the study. The study population comprised 30 patients with CHF who had been treated with digoxin and whose biochemical data were available in the hospital records.

Data collection methods

Relevant data on patient demographics, medical history, and drug administration were extracted. The demographic distribution of the sample population was tabulated to represent the gender and age distribution. Patients were categorized based on gender (male and female) and age (less than 50 years and 50 years or more). For patients treated with digoxin, several renal function indicators were examined both before and after the treatment. These indicators included eGFR, creatinine level, BUN, serum albumin, and urine levels. Apart from the renal function indicators, liver enzymes, such as aspartate aminotransferase (AST) and alanine aminotransferase (ALT), and other parameters like myoglobin, troponin, creatine kinase, and lactic dehydrogenase (LHD), were analyzed. Also, parameters like the admission period, bilirubin levels, platelet count, and hemoglobin levels were evaluated.

Outcome measures

The primary outcome measure was the change in estimated GFR (eGFR) after digoxin treatment. Secondary outcomes included changes in other renal markers such as creatinine, BUN, albumin, and urine levels. Additionally, liver enzymes and other relevant parameters were analyzed.

Data analysis

The collected data were analyzed using IBM SPSS Statistics for Windows, Version 26.0 (Released 2019; IBM Corp., Armonk, New York, United States). The initial analysis generated descriptive statistics such as means, standard deviations, and percentages to summarize the sample characteristics. Paired t-tests were utilized to compare renal function markers like eGFR, creatinine, BUN, albumin, and urine levels before and after digoxin treatment. The t-tests assessed whether changes from baseline were statistically significant at the p<0.05 level. Comparisons of digoxin by gender and age categories were performed using independent sample t-tests. This evaluated whether digoxin's effects differed significantly between subgroups.

Ethical consideration

The study complied with the ethical guidelines provided by various Saudi Arabian and global health organizations. Ethical approval was obtained from the Asir Health Region Directorate (approval number: H-06-B-091). All records were securely stored with limited access.

## Results

Table 1 depicts selected characteristics of the population and their corresponding frequencies and percentages. Gender-wise, the population consists of a higher percentage of males (63.3%) compared to females (36.7%), with 19 males and 11 females. Regarding the age distribution, most of the population falls into the "50 years and older" category, accounting for 76.7% of the total, translating to 23 individuals. In contrast, only 23.3% of the population, or seven individuals, are "less than 50 years."

**Table 1:** Demographic characteristics of the study sample (n = 30)

Selected Characteristics		Frequency	Percent
Gender	Male	19	63.3
	Female	11	36.7
Age	Less than 50 years	7	23.3
	50 years and more	23	76.7

Table 2 shows the impact of digoxin on renal functions in patients with CHF. The variables under examination included eGFR, creatinine level, BUN, serum albumin, and urine levels, measured before and after treatment with digoxin. The mean eGFR significantly decreased from 65.4 ± 8.9 mL/min/1.73 m² before treatment to 57.7 ± 7.8 mL/min/1.73 m² after treatment, with a p-value of 0.001, indicating a statistically significant change. Conversely, the mean creatinine levels increased from 1.61 ± 0.63 mg/dL before treatment to 1.84 ± 0.84 mg/dL after treatment, but this change was not statistically significant (p = 0.241). Similarly, there were increases in the mean levels of BUN, serum albumin, and urine levels post-treatment (from 7.15 ± 2.55 mg/dL to 7.66 ± 2.65 mg/dL, 2.51 ± 1.61 g/dL to 3.07 ± 1.21 g/dL, and 7.46 ± 2.06 mg/dL to 7.99 ± 2.50 mg/dL, respectively). Still, none of these changes were statistically significant (p = 0.448, p = 0.135, and p = 0.377, respectively). Overall, the results indicate that digoxin treatment in patients with CHF led to a significant reduction in eGFR but did not have a statistically significant impact on creatinine, BUN, serum albumin, or urine levels.

**Table 2:** The effect of digoxin on renal functions in patients with CHF ^*^P < 0.05 (significant) eGFR: estimated glomerular filtration rate; BUN: blood urea nitrogen; N: number; SD: standard deviation; CHF: congestive heart failure

Measurements	Test	N	Mean	SD	T	P-value
eGFR	Before	30	65.4	8.9	3.532	0.001*
	After	30	57.7	7.8		
Creatinine level	Before	30	1.61	0.63	1.184	0.241
	After	30	1.84	0.84		
BUN	Before	30	7.15	2.55	0.765	0.448
	After	30	7.66	2.65		
Serum albumin	Before	30	2.51	1.61	1.517	0.135
	After	30	3.07	1.21		
Urine levels	Before	30	7.46	2.06	0.889	0.377
	After	30	7.99	2.50		

Table 3 illustrates the changes in various renal function indicators and other parameters before and after digoxin intake. Each parameter was assessed in 30 subjects, and the mean, SD, t-value, and p-value were calculated for each. The mean AST level increased significantly from 34.50 ± 11.60 U/L before digoxin intake to 53.80 ± 14.61 U/L after (p = 0.002). Similarly, the mean ALT level increased significantly from 38.47 ± 12.58 U/L before intake to 55.33 ± 17.64 U/L after (p = 0.013). Creatine kinase levels increased significantly post-digoxin intake, from 117.73 ± 22.52 U/L to 133.93 ± 15.84 U/L (p = 0.012). Hemoglobin levels, however, decreased significantly from 12.83 ± 1.40 g/dL to 12.05 ± 1.36 g/dL (p = 0.034).

**Table 3:** Renal functions and indicators before and after digoxin intake ^*^P < 0.05 (significant) eGFR: estimated glomerular filtration rate; N: number; SD: standard deviation; AST: aspartate aminotransferase; ALT: alanine aminotransferase; LHD: lactic dehydrogenase; Ca++: calcium; Cl: chloride

Items	Test	N	Mean	SD	T	P-value
AST	Before	30	34.50	11.60	3.322	0.002*
	After	30	53.80	14.61		
ALT	Before	30	38.47	12.58	2.561	0.013*
	After	30	55.33	17.64		
Myoglobin	Before	30	53.53	16.64	0.563	0.576
	After	30	51.13	16.41		
Troponin	Before	30	0.18	0.08	0.071	0.943
	After	30	0.18	0.07		
Creatine kinase	Before	30	117.73	22.52	2.590	0.012*
	After	30	133.93	15.84		
LHD	Before	30	232.47	23.63	0.782	0.437
	After	30	244.97	19.07		
Admission period/days	Before	30	12.87	4.60	0.000	1.0
	After	30	12.87	4.60		
Bilirubin	Before	30	1.00	0.33	0.787	0.435
	After	30	1.07	0.29		
Platelets	Before	30	137.03	26.07	0.404	0.687
	After	30	132.33	23.96		
Hemoglobin	Before	30	12.83	1.40	2.172	0.034*
	After	30	12.05	1.36		
Thyroid function	Before	30	4.2	2.4	1.1	0.07
	After	30	4.3	2.3		
K^+^ level	Before	30	4.1	2.1	1.2	0.1
	After	30	4.2	2.2		
Ca ++	Before	30	2.4	0.5	1.0	0.12
	After	30	2.3	0.4		
Cl	Before	30	98	2.4	1.1	0.09
	After		98.2	2.3		

Other parameters, including myoglobin, troponin, lactic dehydrogenase (LHD), admission period, bilirubin, platelets, thyroid function, potassium (K+) levels, calcium (Ca++) levels, and chloride (Cl) levels, showed no statistically significant changes post-digoxin intake (p>0.05). Myoglobin levels changed from 53.53 ± 16.64 ng/mL to 51.13 ± 16.41 ng/mL (p=0.576), and troponin levels remained stable at 0.18 ± 0.08 ng/mL before and 0.18 ± 0.07 ng/mL after intake (p = 0.943). LHD levels changed from 232.47 ± 23.63 U/L to 244.97 ± 19.07 U/L (p = 0.437), while bilirubin levels increased slightly from 1.00 ± 0.33 mg/dL to 1.07 ± 0.29 mg/dL (p = 0.435). Platelet count decreased slightly from 137.03 ± 26.07 ×10^9/L to 132.33 ± 23.96 ×10^9/L (p = 0.687), and the admission period remained constant at 12.87 ± 4.60 days (p = 1.0). Thyroid function, K+ levels, Ca++ levels, and Cl levels all showed slight changes, but none were statistically significant (p = 0.07, p = 0.1, p = 0.12, and p = 0.09, respectively). Results indicate that digoxin intake significantly increased AST, ALT, and creatine kinase levels and decreased hemoglobin levels. However, it did not considerably impact myoglobin, troponin, LHD, admission period, bilirubin, platelets, thyroid function, K+ levels, Ca++ levels, or Cl levels.

Table 4 compares the effects of digoxin on kidney function between male and female CHF patients. The mean, SD, t-value, and p-value for each parameter were calculated for both genders. There were no significant differences between males and females in the levels of eGFR, creatinine, BUN, serum albumin, urine levels, AST, myoglobin, troponin, creatine kinase, LHD, admission period, bilirubin, platelets, and hemoglobin (p>0.05 for all). Specifically, the mean eGFR for males was 66.05 ± 7.59 mL/min/1.73 m²; for females, it was 64.18 ± 8.46 mL/min/1.73 m² (p = 0.538). Creatinine levels were identical for both genders at 1.84 mg/dL, with an SD of 0.81 for males and 0.93 for females (p = 0.986). BUN levels were 7.62 ± 2.48 mg/dL for males and 7.74 ± 3.04 mg/dL for females (p = 0.911). Serum albumin levels were 2.97 ± 1.44 g/dL for males and 3.24 ± 1.21 g/dL for females (p = 0.555). Urine levels were 7.77 ± 2.62 mg/dL for males and 8.37 ± 2.37 mg/dL for females (p = 0.534). AST levels were 53.79 ± 13.93 U/L for males and 53.82 ± 16.42 U/L for females (p = 0.996). Myoglobin levels were 51.11 ± 15.62 ng/mL for males and 51.18 ± 18.49 ng/mL for females (p = 0.990). Troponin levels were 0.17 ± 0.07 ng/mL for males and 0.19 ± 0.07 ng/mL for females (p = 0.429). Creatine kinase levels were 137.05 ± 21.87 U/L for males and 128.55 ± 22.78 U/L for females (p = 0.540). LHD levels were 246.42 ± 26.93 U/L for males and 242.45 ± 24.29 U/L for females (p = 0.794). The admission period was 12.74 ± 4.74 days for males and 13.09 ± 4.57 days for females (p = 0.843). Bilirubin levels were 1.06 ± 0.30 mg/dL for males and 1.08 ± 0.29 mg/dL for females (p = 0.833). Platelet counts were 130.32 ± 28.77 ×10^9/L for males and 135.82 ± 23.65 ×10^9/L for females (p = 0.747). Hemoglobin levels were 12.01 ± 1.56 g/dL for males and 12.13 ± 0.97 g/dL for females (p = 0.825). ALT was the only parameter that showed a significant difference between genders, with 49.26 ± 17.30 U/L levels for males and 65.82 ± 13.13 U/L for females (p = 0.011). Overall, the results indicate that digoxin intake had similar effects on kidney function and other parameters in both male and female CHF patients, except for ALT levels, which were significantly higher in females.

**Table 4:** Comparison between males and females in the effect of digoxin on kidney function in CHF patients ^*^P < 0.05 (significant) eGFR: estimated glomerular filtration rate; BUN: blood urea nitrogen; N: number; SD: standard deviation; AST: aspartate aminotransferase; ALT: alanine aminotransferase; LHD: lactic dehydrogenase; Ca++: calcium; Cl: chloride; CHF: congestive heart failure

Items	Gender	N	Mean	SD	T	P-value
eGFR	male	19	66.05	7.59	0.624	0.538
	female	11	64.18	8.46		
Creatinine level	male	19	1.84	0.81	0.018	0.986
	female	11	1.84	0.93		
BUN	male	19	7.62	2.48	0.113	0.911
	female	11	7.74	3.04		
Serum albumin	male	19	2.97	1.44	0.597	0.555
	female	11	3.24	1.21		
Urine levels	male	19	7.77	2.62	0.630	0.534
	female	11	8.37	2.37		
AST	male	19	53.79	13.93	0.005	0.996
	female	11	53.82	16.42		
ALT	male	19	49.26	17.30	2.741	0.011*
	female	11	65.82	13.13		
Myoglobin	male	19	51.11	15.62	0.012	0.990
	female	11	51.18	18.49		
Troponin	male	19	0.17	0.07	0.802	0.429
	female	11	0.19	0.07		
Creatine kinase	male	19	137.05	21.87	0.620	0.540
	female	11	128.55	22.78		
LHD	male	19	246.42	26.93	0.264	0.794
	female	11	242.45	24.29		
Admission period/days	male	19	12.74	4.74	0.200	0.843
	female	11	13.09	4.57		
Bilirubin	male	19	1.06	0.30	0.213	0.833
	female	11	1.08	0.29		
Platelets	male	19	130.32	28.77	0.325	0.747
	female	11	135.82	23.65		
Hemoglobin	male	19	12.01	1.56	0.224	0.825
	female	11	12.13	0.97		

Table 5 compares the effect of digoxin on kidney function in CHF patients according to age. The parameters measured included eGFR, creatinine level, BUN, serum albumin, urine levels, AST, ALT, myoglobin, troponin, creatine kinase, LHD, admission period, bilirubin, platelets, and hemoglobin. The patients were grouped into two categories: those less than 50 years old and those 50 years and older. Most parameters showed no significant difference between the two age groups. The mean eGFR was 68.71 ± 6.97 mL/min/1.73 m² for patients under 50 years and 64.35 ± 7.93 mL/min/1.73 m² for patients 50 years and older (p = 0.202). The creatinine levels were 1.36 ± 0.30 mg/dL for patients under 50 years and 1.99 ± 0.90 mg/dL for patients 50 years and older (p = 0.082). The serum albumin levels were 3.06 ± 1.07 g/dL for patients under 50 years and 3.07 ± 1.27 g/dL for patients 50 years and older (p = 0.975). The urine levels were 8.51 ± 1.54 mg/dL for patients under 50 years and 7.83 ± 2.74 mg/dL for patients 50 years and older (p = 0.536). The AST levels were 55.29 ± 15.38 U/L for patients under 50 years and 53.35 ± 14.69 U/L for patients 50 years and older (p = 0.765). The ALT levels were 58.14 ± 18.53 U/L for patients under 50 years and 54.48 ± 17.70 U/L for patients 50 years and older (p = 0.639). The myoglobin levels were 61.29 ± 15.12 ng/mL for patients under 50 years and 48.04 ± 15.80 ng/mL for patients 50 years and older (p = 0.060). The troponin levels were 0.16 ± 0.07 ng/mL for patients under 50 years and 0.18 ± 0.07 ng/mL for patients 50 years and older (p = 0.474). The creatine kinase levels were 123.57 ± 23.09 U/L for patients under 50 years and 137.09 ± 26.74 U/L for patients 50 years and older (p = 0.392). The LHD levels were 245.43 ± 23.62 U/L for patients under 50 years and 244.83 ± 22.11 U/L for patients 50 years and older (p = 0.972). The admission period was 11.71 ± 4.39 days for patients under 50 years and 13.22 ± 4.70 days for patients 50 years and older (p = 0.459). The bilirubin levels were 1.17 ± 0.29 mg/dL for patients under 50 years and 1.03 ± 0.29 mg/dL for patients 50 years and older (p = 0.285). The platelet counts were 133.29 ± 26.51 ×10^9/L for patients under 50 years and 132.04 ± 24.73 ×10^9/L for patients 50 years and older (p = 0.949). The hemoglobin levels were 11.61 ± 1.10 g/dL for patients under 50 years and 12.19 ± 1.42 g/dL for patients 50 years and older (p = 0.336). BUN was the only parameter that showed a significant difference between the two age groups, with 5.59 ± 2.72 mg/dL levels for patients under 50 years and 8.30 ± 2.33 mg/dL for patients 50 years and older (p = 0.015). The results indicate that digoxin intake had similar effects on kidney function and other parameters in CHF patients, regardless of age, except for BUN levels, which were significantly higher in patients 50 and older.

**Table 5:** Comparison of the effect of digoxin on kidney function in CHF patients according to age ^*^P < 0.05 (significant) eGFR: estimated glomerular filtration rate; BUN: blood urea nitrogen; N: number; SD: standard deviation; AST: aspartate aminotransferase; ALT: alanine aminotransferase; LHD: lactic dehydrogenase; Ca++: calcium; Cl: chloride; CHF: congestive heart failure

Items	Age	N	Mean	SD	T	P-value
eGFR	less than 50 years	7	68.71	6.97	1.308	0.202
	50 years and more	23	64.35	7.93		
Creatinine level	less than 50 years	7	1.36	0.30	1.807	0.082
	50 years and more	23	1.99	0.90		
BUN	less than 50 years	7	5.59	2.72	2.596	0.015
	50 years and more	23	8.30	2.33		
Serum albumin	less than 50 years	7	3.06	1.07	0.032	0.975
	50 years and more	23	3.07	1.27		
Urine levels	less than 50 years	7	8.51	1.54	0.626	0.536
	50 years and more	23	7.83	2.74		
AST	less than 50 years	7	55.29	15.38	0.302	0.765
	50 years and more	23	53.35	14.69		
ALT	less than 50 years	7	58.14	18.53	0.475	0.639
	50 years and more	23	54.48	17.70		
Myoglobin	less than 50 years	7	61.29	15.12	1.959	0.060
	50 years and more	23	48.04	15.80		
Troponin	less than 50 years	7	0.16	0.07	0.725	0.474
	50 years and more	23	0.18	0.07		
Creatine kinase	less than 50 years	7	123.57	23.09	0.870	0.392
	50 years and more	23	137.09	26.74		
LHD	less than 50 years	7	245.43	23.62	0.035	0.972
	50 years and more	23	244.83	22.11		
Admission period/days	less than 50 years	7	11.71	4.39	0.751	0.459
	50 years and more	23	13.22	4.70		
Bilirubin	less than 50 years	7	1.17	0.29	1.089	0.285
	50 years and more	23	1.03	0.29		
Platelets	less than 50 years	7	133.29	26.51	0.064	0.949
	50 years and more	23	132.04	24.73		
Hemoglobin	less than 50 years	7	11.61	1.10	0.978	0.336
	50 years and more	23	12.19	1.42		

## Discussion

The current research study aimed to evaluate the potential effects of digoxin on renal functions in patients with CHF. The paired sample t-test was utilized, and the study demonstrated a significant decrease in the level of eGFR following digoxin intake. This aligns with the findings of Shah et al., who reported a greater rate of decline in eGFR in patients on digoxin, suggesting digoxin's potential renal toxicity [3]. Past studies found a negative correlation between digoxin and eGFR, reinforcing that digoxin is primarily eliminated through the kidneys [7,8].

Regarding creatinine levels, the study found no statistically significant differences in patients with CHF before and after digoxin intake. This aligns with Testani et al., who found no significant difference in creatinine levels when digoxin was administered to patients with CHF [4]. On the contrary, Mutlu et al. reported elevated creatinine levels in neonates treated with digoxin after delivery [9].

Concerning BUN, there were statistically significant differences in pre- and post-digoxin intake, which is consistent with the findings of Ibrahim et al. The study found no statistically significant differences in serum albumin levels before and after digoxin intake [10]. Shen et al. had similar findings, with a significant relationship between digoxin intake and serum albumin [7].

The study found no statistically significant differences in urine alkalinity levels in patients with CHF before and after digoxin intake. Regarding AST and ALT, there were statistically significant differences in patients with CHF before and after digoxin intake, indicating a negative effect of digoxin on these liver enzymes. This contrasts with another study that argued that digoxin is not associated with AST or ALT elevations [11].

The study also found no statistically significant differences concerning levels of myoglobin and troponin. However, a significant relationship was found between creatine kinase levels and digoxin intake. This aligns with the findings of another study that digoxin induces a concentration-dependent release of creatine kinase [12]. The study found no significant differences in digoxin intake between LHD and bilirubin. This is consistent with other studies [13,14]. Finally, no significant differences were found in relation to digoxin intake for platelets, aligning with another study that reported that digoxin did not affect platelet aggregation [15].

The current study found mixed results regarding the effect of digoxin on renal and liver functions in patients with CHF. Some variables showed significant changes post-digoxin intake, while others did not. These findings highlight the need for further investigations to fully understand the impact of digoxin on different biochemical parameters in patients with CHF.

Study limitations

One limitation of this study is the small sample size of 30 patients. With such a limited number of participants, the ability to generalize the results to the overall population is reduced. A larger sample would have increased the power and accuracy of the statistical analysis. Additionally, the study only assessed patients over a short period after digoxin intake. The long-term effects on renal function and other parameters beyond the study period are unknown. A longer follow-up would have provided more insight into potential lasting impacts. The study also did not control for other medications or comorbidities that patients may have been taking or experiencing concurrently. Other pharmacological or disease factors could have influenced the changes observed, introducing confounding variables. A more controlled experimental design would have limited these potential confounds. Lastly, the study used a pre-post assessment design without a control group. Changes seen after digoxin may not fully be attributable to the drug itself, as other temporal factors could account for some of the differences observed. Including a control arm would have strengthened conclusions about digoxin's effects.

## Conclusions

This retrospective study provides preliminary evidence that digoxin treatment may adversely affect renal function in CHF patients, as indicated by a statistically significant reduction in eGFR. However, digoxin did not appear to substantially impact other markers of renal function like creatinine, BUN, albumin, and urine levels. Effects on liver enzymes were mixed, with significant increases seen in AST, ALT, and creatine kinase but no change in other parameters. Hemoglobin decreased significantly as well. Comparisons by gender and age did not reveal significant differences in digoxin's effects, suggesting impacts on renal function are not strongly modified by demographic factors.

Overall, while these initial results suggest potential renal-related risks of digoxin therapy in CHF patients, the small retrospective design restricts definitive conclusions. Additional prospective research with more extensive, diverse samples, control groups, standardized data collection, and more comprehensive kidney function measures is warranted to clarify digoxin's effects on renal physiology. Detailed studies can help establish appropriate monitoring and precautions when using digoxin in patients with CHF and concomitant renal dysfunction. However, the current evidence is not sufficiently robust to deviate from established digoxin dosing guidelines in this population.
